# Supplementary material for: Age‐Associated Decline in Autophagy Pathways in the Retinal Pigment Epithelium and Protective Effects of Topical Trehalose in Light‐Induced Outer Retinal Degeneration in Mice
Source: Aging Cell. 2025 Apr 28;24(7):e70081. doi: 10.1111/acel.70081 (PMC12266760; doi:10.1111/acel.70081)
Supplement: Supplementary file 1 — Appendix S1. [file ACEL-24-e70081-s002.docx]

**SUPPLEMENTARY METHODS**

**Mouse RPE isolation**

Mouse eyes were enucleated and cleaned using angled scissors to remove any remaining connective tissue. After removing the cornea and lens, eyecups were incubated at 37°C in hyaluronidase (Sigma-Aldrich) for 45 min, and in HBSS buffer with 10 mM HEPES for a further 30 min before retinas were removed via incision. Subsequently, eyecups were incubated at 37°C in trypsin/EDTA for 45 min before being transferred into HBSS with 20% heat-inactivated FCS. Gently shaking facilitated the detachment of RPE sheets, which were incubated in trypsin/EDTA for 1 min to form single-cell suspensions. The cells were either processed for RNA extraction or resuspended in cell culture medium for cultivation and subsequent treatments as specified.

**Primary murine RPE cell culture**

Isolated murine RPE cells were resuspended in alpha MEM supplemented with 1% N1 Medium Supplement (Sigma-Aldrich), 1% L-glutamine, 1% penicillin–streptomycin, 1% nonessential amino acid solution (Thermo Fisher Scientific), 20 μg/l hydrocortisone (Sigma-Aldrich), 250 mg/l taurine (Sigma-Aldrich), 0.013 μg/l triiodo-thyronin (Sigma-Aldrich), and 5% FCS. The cells were seeded to laminin (Sigma-Aldrich)-precoated 24-well cell culture plates, Seahorse XFp Cell Culture Plates (Agilent Technologies), or 16-well chambered cover glass (Thermo Fisher Scientific), at a density of 25,000/cm^2^. Serum was withdrawn after one week of incubation, and the cells were kept in serum-free condition for an additional week for specified treatments. Cell culture media were refreshed twice weekly.

**Cytotoxicity assay**

RPE cells were incubated with different concentrations of trehalose (6.25, 12.5, and 25 mM, Sigma-Aldrich, PHR1344) for 24 h. Cell culture supernatants were collected for assessment using a Lactate Dehydrogenase (LDH) cytotoxicity kit (Abcam).

**Seahorse metabolic assay**

Effects of trehalose and/or H_2_O_2_ on mitochondrial respiratory in RPE cells were assessed using Mito Stress tests on a Seahorse Cell Metabolic Analyzer (Agilent Technologies). Seahorse XFp cell culture miniplates, sensor cartridges, and all reagents were from Agilent Technologies. Cells were incubated in Seahorse XF DMEM containing 25 mM glucose, 1 mM pyruvate, and 2 mM glutamine in 37°C incubator without CO_2_ for 45 min. Oligomycin (1 μM), Carbonyl cyanide-p-trifluoromethoxyphenylhydrazone (FCCP, 0.5 μM) and antimycin A/rotenone (1 μM) were injected where indicated. OCR (pmol O_2_/min) was measured in real-time and normalized by total protein analyzed using a BCA assay. OCR parameters were calculated using the following formulae: nonmitochondrial respiration (NMR, minimum OCR after antimycin A/rotenone injection), basal respiration (BR, difference between OCR before oligomycin and NMR), maximal respiration (MR, difference between maximum OCR after FCCP injection and NMR), spare respiratory (SR, difference between MR and BR), ATP production (difference between OCR before oligomycin injection and minimum OCR after Oligomycin).

**Autophagy flux measurement**

The formation of autophagosome and autolysosome in RPE cells was monitored through LC3B localization using a Premo™ Autophagy Tandem sensor Kit (Thermo Fisher Scientific), which detects LC3B positive, neutral pH autophagosomes in green fluorescence (GFP) and LC3B positive, acidic pH autolysosome in red fluorescence (RFP). The reagent was added to RPE cells (40 particles/cell). After 24 h, cells were treated with trehalose (12.5 mM) for an additional 16 h. Subsequently, 50 nM of LysoTracker Deep Red (Thermo Fisher Scientific) was applied to the cells for 30 min for lysosome staining. After washing with PBS and counter-staining with Hoescht 33342 (Thermo Fisher Scientific), cells were imaged live on a Leica SP5II confocal laser scanning microscope. Z-stack images of cells were acquired using 1-µm step size and visualized using maximal intensity projections. LC3B-positive vacuoles were quantified using Fiji.

**Mitochondrial superoxide staining**

To detect mitochondrial superoxide in primary murine RPE cells treated with trehalose and/or H_2_O_2_, MitoSOX Red (Thermo Fisher Scientific) was added to the cells at a final concentration of 5 μM for 10 min. After washing with HBSS, cells were stained with Hoechst 33342 and observed using the confocal microscope.

**Raman spectroscopy**

Raman spectroscopy was carried out via inVia Qontor Raman confocal spectrometer with a Leica DMLM microscope and a 785 nm excitation laser. Calibration was conducted on a silicon wafer. For sample preparation, 2 μL of each supernatant was deposited on a glass-aluminium slide and air-dried for 30 min at room temperature. Spectral data were acquired at 55 mW laser power, × 20 objective lens and a 1200 L/mm grating. A typical Raman spectrum was collected in the fingerprint region (750–1750 cm⁻¹) with 3 accumulations with 3 seconds each. A total of 50 spectra per sample were collected through a 5 × 5 Raman mapping scan with 5-μm step intervals. Data acquisition, cosmic ray correction and baseline adjustment were processed using WiRE software (Renishaw). Standard normal variate (SNV) normalization was performed using a Python 3.7 script. Spectral barcoding was conducted by applying the Savitzky-Golay filter to compute second derivative transformations, using a window size of 21 and a polynomial order of 2.

Spectral barcodes and plots were generated for known controls and compared with unknown samples. Negative controls (RPE/choroidal or retinal lysates) exhibited two predominant peaks in the fingerprint region: 1662 cm⁻¹ (nucleic acid) and 1670 cm⁻¹ (amide I) (Figure S7a). Positive controls (lysates spiked with 1 mM trehalose in water) presented spectral features consistent with pure trehalose, including bands at 1002 cm⁻¹ (phenylalanine), 1030 cm⁻¹ (C-H bending, ν(CC) skeletal), 1321 cm⁻¹ (amide III (α-helix)), 1322 cm⁻¹ (CH₃-CH₂ twisting), 1323 cm⁻¹ (CH deformation), 1464 cm⁻¹ (Fermi interaction δ(CH₂) and γ(CH₂)), and 1555 cm⁻¹ (amide II).

**Light-induced retinal degeneration**

Mice were dark-adapted overnight and administered intraperitoneally with 100 µL of 2% FL (Huddersfield Pharmacy Specials) in sterile stilled water. 3 min after the injection, light was delivered at the centre of the left eye at an intensity of 18 kLux for a one-time exposure of 5 min, guided by the fundus camera of a Micron IV device (Phoenix Research Labs). The right eye was left as a control. The experimenter performing the light challenge was blinded to the treatment conditions. Following light challenge, mice were kept under normal lighting conditions.

**Fundoscopy and optical coherence tomography**

Pupils were dilated using topical eyedrops containing 1% tropicamide and 2.5% phenylephrine, and the mice were anaesthetized using 2% isoflurane inhalation. The Micron IV retinal imaging microscope (Phoenix Research Laboratories) was used to capture OCT scans and brightfield fundal images, with a gain of 3 dB and an FPS of 15. OCT scans were taken in 30-degree increments, centered at the optic disc. The ONL thickness was measured on each OCT image across a 400-µm span, beginning 100 µm from either side of the optic nerve head (ONH), resulting in a total of 16 measurements per eye to calculate an average. An independent investigator conducted the measurements in a blinded manner.

**Immunohistochemistry and fluorescence staining**

To examine the expression of autophagy and lysosome-related proteins in mouse retinal sections, eyes were enucleated and serial cryosections of 12-µm thickness were prepared using a cryostat. Sections were fixed with 4% paraformaldehyde (PFA) for 15 min and washed thrice before blocking with 5% bovine serum albumin (BSA), 5% normal donkey serum (NDS) and 0.2% Triton X-100 in PBS. Sections were stained overnight at 4^o^C with primary antibodies diluted in staining buffer (1% BSA in PBS), containing rabbit anti-LC3B (1:1000, Abcam, ab5152), rat anti-LAMP1 (1:50, Santa Cruz Biotechnology, sc-19992), or rabbit anti- p62 (1:1000, Sigma-Aldrich, P0067). After washing, samples were incubated with donkey anti-rabbit IgG-Alexa Fluor (AF)488 or anti-rat IgG-AF555 (1:400, Thermo Fisher Scientific) for 1 h. Slides were then counter-stained with Hoescht 33342. After mounting with anti-fade fluorescence mounting medium (Abcam), samples were imaged with a Leica SP5II confocal laser scanning microscope focused on the RPE region of interest. Mean fluorescence intensity (MFI) was quantified using Fiji.

To prepare RPE/choroid wholemounts for assessment of RPE morphology, enucleated eyes were dissected and RPE-choroid-sclera isolated. Tissues were fixed in 4% PFA for 1 h, washed in PBS, and blocked and permeabilized with 5% BSA, 5% NDS, and 0.3% Tween-20 for 2 h. Tissues were then incubated with Phalloidin-AF555 (1:20 in PBS, New England Biolabs) for 30 min to stain F-actin. After washing and staining with Hoescht 33342, samples were mounted and imaged using the confocal microscope.
