# Supplementary material for: Age‐Associated Decline in Autophagy Pathways in the Retinal Pigment Epithelium and Protective Effects of Topical Trehalose in Light‐Induced Outer Retinal Degeneration in Mice
Source: Aging Cell. 2025 Apr 28;24(7):e70081. doi: 10.1111/acel.70081 (PMC12266760; doi:10.1111/acel.70081)
Supplement: Supplementary file 8 — Appendix S8. [file ACEL-24-e70081-s007.docx]

Data S1: Human RNA-seq DEGs

Data S2: Human RNA-seq DEGs in autophagy-lysosome pathway categories

Data S3: Mouse RNA-seq raw counts, batch-corrected and normalized counts

Data S4: Mouse RNA-seq DEGs in autophagy-lysosome pathway categories

Data S5: Overlapped DEGs between aged human RPE/cho and mouse RPE
